# White Blood Cell Proportions Are Associated With Response to Psychosocial Therapy in Young People at Ultra-High Risk for Psychosis

**DOI:** 10.1016/j.bpsgos.2025.100546

**Published:** 2025-06-03

**Authors:** Lauren F. Barker, Allan F. McRae, Hok Pan Yuen, Anjali K. Henders, Leanne M. Wallace, Tian Lin, Christina Phassouliotis, Jessica Spark, Melissa Kerr, Enda M. Byrne, G. Paul Amminger, Barnaby Nelson, Naomi R. Wray, Patrick D. McGorry

**Affiliations:** a Institute for Molecular Bioscience, The University of Queensland, Saint Lucia, Queensland, Australia; b Orygen, Parkville, Victoria, Australia; c Centre for Youth Mental Health, The University of Melbourne, Melbourne, Victoria, Australia; d Child Health Research Center, The University of Queensland, Saint Lucia, Queensland, Australia; e Department of Psychiatry, University of Oxford, Oxford, United Kingdom

**Keywords:** Clinical trial, DNA methylation, Gene expression, Lymphocytes, Neutrophil-lymphocyte ratio, Remission

## Abstract

**Background:**

White blood cell (WBC) counts, DNA methylation, and gene expression are reported to be associated with psychosis. However, it is not known whether these associations precede the onset of psychosis or whether they are relevant for the stratification of psychosis risk in clinically high-risk individuals. The STEP (Staged Treatment in Early Psychosis) clinical trial evaluated the effectiveness of a sequential intervention strategy for preventing psychosis in a cohort of young people at ultra-high risk (UHR) of psychosis. Participants were assessed for remission of UHR status after up to 6 months of treatment with psychosocial therapy.

**Methods:**

Cell-type proportions estimated from whole-blood DNA methylation samples (*N* = 91) were used to test for associations between WBC proportions at trial baseline and remission of UHR status (31 remitters, 60 nonremitters), including at which step of the trial remission occurred. DNA methylome-wide association and differential expression analyses were conducted to test for associations with remission of UHR status.

**Results:**

Baseline lymphocyte cell proportions (odds ratio [OR], 0.23; 95% CI, 0.07–0.62; *p* = 9.2 × 10^−3^) and neutrophil-lymphocyte ratio (OR, 2.9; 95% CI, 1.37–7.46; *p* = .012) were significantly associated with remission status. There were suggestive associations between specific cell types and the timing of remission during the trial; however, these did not survive correction for multiple testing. No methylation probes or differentially expressed genes were associated with remission status when cell-type proportions were included as covariates.

**Conclusions:**

Our results indicate the potential importance of WBCs for further stratification of psychosis risk in UHR individuals and reinforce the importance of routine collection of WBC data for future clinical trials.

## Plain language summary

The Staged Treatment in Early Psychosis clinical trial evaluated the effectiveness of a sequential intervention strategy for preventing psychosis in a cohort of young people at ultra-high risk (UHR) of psychosis. In a follow-up analysis of this trial, we used DNA methylation data to estimate white blood cell (WBC) proportions in trial participants at baseline. We report that low lymphocyte proportion and high neutrophil-lymphocyte ratio at baseline were associated with remission of UHR status after treatment with psychosocial therapy. We conclude that WBC proportions could be used in predictive modelling for psychosis risk together with other risk factors.

Inflammation and the immune system are becoming increasingly implicated in the etiology and pathogenesis of schizophrenia (SCZ) and related psychotic disorders. As key components of the immune system, white blood cells (WBCs) in peripheral blood samples are routinely measured in clinical settings to assess infection, illness, and immune function (1). In keeping with the hypothesis that immune dysfunction and inflammation have contributing roles in the pathogenesis of SCZ and psychosis, associations between both total WBC count and counts of individual WBCs have frequently been observed in these disorders (2). A meta-analysis of published WBC differential (WBCD) results found that neutrophil and monocyte counts were consistently significantly higher for SCZ cases compared with healthy controls (3). Neutrophil and monocyte counts were likewise significantly elevated in first-episode psychosis (FEP), despite there being fewer studies to draw on. In the meta-analysis, lymphocytes were not found to be significantly associated with SCZ or FEP, despite previous associations with reduced lymphocyte counts (4, 5, 6). However, there is increasing evidence that lymphocytes may still be highly relevant for identifying potential WBC-based markers of disease, due to their role in the adaptive (or acquired) immune response. Comparing the proportion of lymphocytes with WBCs involved in the innate, inflammatory immune response, such as neutrophils and monocytes, may provide information about the relative activity of each system (7).

The neutrophil-lymphocyte ratio (NLR) is one such comparison that is widely used as a marker of systemic inflammation (7). A higher NLR can be observed in several clinical situations such as bacteremia (8), stroke (9), and heart attack (10) and is also associated with mortality, both overall and from more specific causes such as heart disease and influenza (11,12). Numerous studies and meta-analyses have identified higher NLR in psychotic disorders, including SCZ and FEP (13, 14, 15). This finding persists regardless of severity of symptoms or phase, including acute relapse and remission, and does not appear to differ between patient subgroups based on phase or severity (15, 16, 17). This suggests that elevated NLR may be a potential marker of psychotic illness in any form as opposed to a marker of specific diagnosis or current psychotic status. However, it should be noted that antipsychotic medications may influence NLR: Cross-sectional and case-control studies have reported mixed results of NLR between medicated and nonmedicated participants (18, 19, 20); however, longitudinal studies of the same participants with FEP and SCZ over time have noted a decrease in NLR after treatment with antipsychotics (21,22).

In addition to cell-based markers of psychosis, numerous studies have also tried to identify molecular markers using data types such as DNA methylation and gene expression. The largest study of DNA methylation in SCZ to date, a meta-analysis of 7 different cohorts, identified that both FEP and SCZ cases displayed differential methylation relative to healthy controls (23). However, FEP cases displayed far fewer differentially methylated sites and smaller effect sizes than the diagnosed SCZ cases. This meta-analysis also identified that many of the SCZ-associated probes were also associated with C-reactive protein, a common clinical marker of inflammation (24).

Psychosis-associated DNA methylation changes may also precede the onset of psychotic symptoms (25, 26, 27, 28), with several studies of animal models implicating prenatal neuroinflammation–induced methylation changes in later onset of SCZ-associated symptoms (29, 30, 31). In humans, neonatal blood samples from individuals who would later be diagnosed with SCZ display differential methylation compared with healthy control individuals. These differentially methylated genes in neonates are differentially expressed in postmortem brain tissue from adults who were diagnosed with SCZ (relative to healthy control individuals) (28), indicating that DNA methylation may be useful as a predictive marker of psychosis.

Gene expression studies have identified a large number of genes that are differentially expressed in SCZ and FEP cases relative to healthy controls (32, 33, 34, 35); however, comparatively fewer studies have focused on individuals at high risk for psychosis. The few studies that have focused on these high-risk cohorts have identified that high-risk individuals typically have less extreme changes to expression profiles than either SCZ or FEP cases compared with healthy controls (36,37). However, it should be noted that these studies have mostly used a candidate gene approach, and despite using whole-blood samples, they did not correct for cell-type composition.

The STEP (Staged Treatment in Early Psychosis) clinical trial aimed to test the effectiveness of a sequential intervention strategy for preventing psychosis in adolescents and young people at ultra-high risk (UHR) of psychosis (38, 39, 40). During the trial, participants received psychosocial therapy and were assessed for remission of UHR status at 2 different time points. In this analysis of the STEP data, we used cell-type proportions derived from DNA methylation samples to test for associations between WBC proportions at trial baseline and remission of UHR status following treatment with psychosocial therapy. We also utilized longitudinal collection of gene expression and DNA methylation samples to investigate potential signals at baseline of subsequent remission, as well as longitudinal changes that may indicate pathways involved in UHR remission.

## Methods and Materials

### Clinical Trial

The STEP clinical trial was designed to evaluate the effectiveness of a sequential intervention strategy for preventing psychosis in young people at UHR of developing psychosis, and results have been reported previously (38, 39, 40). Enrollment occurred between April 2016 and January 2019 and was approved by the Melbourne Health Human Research Ethics Committee. Briefly, the study used a clinical staging model, the first 2 steps of which comprised 2 different forms of psychosocial therapy. Participants received open-label support and problem solving (SPS) for the first 6 weeks. Participants who did not meet remission criteria after step 1 were randomized to either SPS or cognitive behavioral case management (CBCM) for the following 20 weeks. Participants were assessed for remission of UHR status at 4 and 6 weeks for step 1 and at 12 and 24 weeks for step 2 and were required to meet remission criteria at both assessment time points to be considered remitters for that step. Individuals were recruited from Orygen's PACE (Personal Assessment and Crisis Evaluation) clinic and 4 headspace youth mental health centers in Melbourne, Australia, and were included in the study if they were 12 to 25 years of age, were able to speak adequate English and provide informed consent, and met the UHR psychosis criteria (38,40). Participants were excluded if they had already had a previous extended psychotic episode (>1 week) or had neurological, developmental, metabolic, or other physical conditions known to cause psychosis. Participants were also excluded if they were currently on antidepressant or antipsychotic medication and were assessed as needing to continue treatment with this medication. Participants were not excluded for having a preexisting psychiatric diagnosis. Participants who did not respond to the first 2 steps of the trial entered a third step of treatment, during which they received CBCM together with either fluoxetine or a placebo. Due to the different modes of treatment in step 3 compared with steps 1 and 2, the analyses in this article are focused solely on remission after steps 1 and 2.

### Assessments

UHR status was established using the Comprehensive Assessment of At-Risk Mental States (CAARMS) (41) and Social and Occupational Functioning Assessment Scale. Participants also completed a self-reported Childhood Trauma Questionnaire (CTQ) (40), as well as the Montgomery–Åsberg Depression Rating Scale (MADRS) (42), Global Functioning Social and Role scales (43), Brief Psychiatric Rating Scale (BPRS) (44), Scale for the Assessment of Negative Symptoms (45), and Davos Assessment of Cognitive Biases Scale (46). Additionally, participants underwent diagnostic testing at baseline to establish whether they met criteria for any psychiatric diagnoses.

STEP trial participants were given the option to consent to participation in genomic analyses. Blood samples from consenting participants were used to generate genomic data, including genotypes, DNA methylation profiles, and gene expression profiles. The bio-cohort who provided 1 or more of these samples did not differ from the main trial cohort on most demographic or clinical measures (Tables S3 and S4). Generation of genomic data was conducted under Human Research Ethics governance of the University of Queensland.

### Preparation of DNA Methylation Data

Methylation profiling was performed on whole-blood samples using the Infinium MethylationEPIC array (version 1), and quality control (QC) and normalization were performed with the R package *meffil*, using recommended parameters (47). Full details of data generation and QC can be found in Supplemental Methods.

### Estimation and Validation of DNA Methylation–Derived Cell Proportions

Cell-type proportions were estimated using the Houseman method (48) as implemented in the *meffil* package. Cell-specific methylation profiles from Reinius *et al.* (49) were used as the reference dataset.

Participants who proceeded to step 3 of the trial were required to have clinical blood tests performed by a pathology service for monitoring safety while on trial medications. These blood tests were performed at 6 and 12 months and included WBCDs. Baseline methylation-derived cell-type proportions were validated using a subset (*n* = 104) of these 6- and 12-month STEP samples, for which both WBCD and DNA methylation–derived cell proportions were available. As the WBCD only measures total lymphocytes, methylation-derived cell proportions from cell types within the lymphocyte category were combined to get an estimated proportion for total lymphocyte cells. Pearson’s correlations were estimated for WBCDs and methylation-derived cell proportions across all cell types, as well as separately for each individual cell type.

### Statistical Analysis

#### Sample Selection

Participants who had a clinical remission status after steps 1 and 2 of the trial were included if they had a valid baseline DNA methylation sample, a genotype sample for inference of genetic ancestry (see Genome-wide genotyping in Supplemental Methods and Figure S1), and nonmissing psychiatric diagnosis, MADRS, and CTQ data at baseline. As selective serotonin reuptake inhibitors (SSRIs) can affect blood cell counts (50), participants were excluded if they reported taking an SSRI or other antidepressant within 30 days of trial baseline (*n* = 1). No participant reported taking antipsychotic medication within the year prior to trial baseline.

#### Univariate Testing of Remission Status

Differences in continuous demographic (age) and clinical (MADRS, CTQ scores) variables between remitters and nonremitters were tested using Wilcoxon-Mann-Whitney tests, as implemented in the R package *coin* (51). For clinical scores containing subdomains (e.g., MADRS, CAARMS), the total score was used (i.e., summed across subdomains). For noncontinuous demographic and clinical variables, independence in distribution between remitters and nonremitters were tested using Pearson’s χ^2^ test. A Bonferroni-adjusted *p* value significance threshold of 3.58 × 10^−3^ was used to determine significance. The same method and significance threshold were used to test for differences between step 1 remitters and step 2 remitters.

Pearson correlations were calculated between numeric demographic variables, clinical scores, and methylation-derived cell proportions and were adjusted for multiple testing using the Benjamini-Hochberg procedure on unique comparisons.

#### Logistic Regression Analyses

Logistic regression analysis was used to assess the association between remission status and methylation-derived cell proportions for the WBCD-validated cell types (lymphocytes, monocytes, neutrophils, and eosinophils) and, in follow-up analyses, for the 4 lymphocyte cell subtypes (CD4+ T cells, CD8+ T cells, B cells, and natural killer cells) and the log NLR (calculated as log[neutrophils/lymphocytes]). Due to collinearity between cell proportions, only 3 of the 4 cell types (lymphocytes, monocytes, and eosinophils) were included. Accordingly, neutrophils were also not included in the sublymphocyte regressions. Cell proportions were first mean centered and scaled by the standard deviation to increase the interpretability of the results. Each set of cell proportions was tested using demographic covariates (equation 1) and demographic and clinical covariates (equation 2) as follows:

(1) RemissionStatus∼Sex+Age+GeneticPC1+GeneticPC2+Lymphocytes+Monocytes+Eosinophils

(2) RemissionStatus∼Sex+Age+GeneticPC1+GeneticPC2+BaselineMADRS+CTQ+HasPsychiatricDiagnosis (Yes / No)+Lymphocytes+Monocytes+Eosinophils

To assess whether timing of remission was associated with cell-type proportions, the above analyses were repeated using pairwise logistic regressions to compare step 1 remitters, step 2 remitters, and nonremitters. *p* Values of association were assessed against thresholds corrected for multiple testing (*p* < .0125 for remission status and *p* < 6.25 × 10^−3^ for the step of remission). See the Supplement for full details of these analyses.

### Methylome-Wide Association Study

Methylome-wide association studies (MWASs) of remission phenotypes were performed using mixed-effect models. MWASs were performed on baseline samples and on a combined dataset of baseline and 6-month samples, the latter of which was performed to identify probes exhibiting changes in DNA methylation levels during the course of remission. Full methods can be found in Supplemental Methods.

### Differential Expression

Differential expression analyses of remission phenotypes were performed using *limma-voom* (52). Differential expression analyses were performed on baseline samples and on a combined dataset of baseline and 6-month samples, the latter of which was performed to identify genes with changes in expression levels during the course of remission. Full methods can be found in Supplemental Methods.

## Results

### Sample Characteristics

DNA methylation–derived cell-type proportions were available for 91 eligible participants at trial baseline, 31 of whom were remitters and 60 of whom were nonremitters. Remitters and nonremitters did not differ significantly on sex or age (Table 1).

**Table 1:** Baseline Demographic and Clinical Characteristics of STEP Remitters and Nonremitters

	Remitter, *n* = 31	Nonremitter, *n* = 60	*p* Value^a^	Missing, *n*
Age, Years	17.4 (3.6)	16.8 (2.8)	.71	0
Female	41.9%	58.3%	.18	0
European Ancestry^b^	83.9%	75.0%	.43	0
Preexisting Psychiatric Diagnosis	51.6%	95.0%	2.3 × 10^−6^	0
Recent SSRI Use^c^	6.5%	15.0%	.32	0
MADRS Total Score	14.8 (9.1)	25.1 (9.3)	1.1 × 10^−5^	0
CAARMS Total Score	15.0 (8.1)	21.9 (7.5)	2.3 × 10^−4^	1
BPRS Total Score	38.0 (6.6)	46.5 (8.5)	4.8 × 10^−6^	0
SANS Total Score	12.5 (9.3)	21.0 (12.1)	6.6 × 10^−4^	0
SOFAS Score	60.3 (10.3)	55.5 (11.4)	.10	0
DACOBS Total Score	160.3 (25.7)	165.5 (26.5)	.40	5
Global Functioning: Social	7.0 (1)	6.3 (1.2)	.0052	0
Global Functioning: Role	6.8 (1.6)	6.3 (1.4)	.062	0
Childhood Trauma Questionnaire Total Score	54.6 (11.2)	62.2 (14.1)	.0081	0

Values are presented as mean (SD) or %.

BPRS, Brief Psychiatric Rating Scale; CAARMS, the Comprehensive Assessment of At-Risk Mental States; DACOBS, Davos Assessment of Cognitive Biases Scale; MADRS, Montgomery–Åsberg Depression Rating Scale; SANS, Scale for the Assessment of Negative Symptoms; SOFAS, Social and Occupational Functioning Assessment Scale; SSRI, selective serotonin reuptake inhibitor; STEP, Staged Treatment in Early Psychosis.

a Bonferroni-adjusted *p* value significance threshold is 3.58 × 10^−3^.

b Genetically inferred ancestry group.

c Reported use within the past 12 months.

Genetic ancestry was estimated for all participants to account for potential ancestry-related bias in methylation-derived cell proportions. Most participants were inferred to be of European ancestry, and the proportion of participants genetically predicted to be European did not differ significantly between remitters and nonremitters (Table 1).

Nonremitters had a more clinically severe profile than remitters, with nonremitters having significantly higher mean baseline scores for depression and attenuated psychotic symptoms, as well as nominally poorer scores for functioning and higher scores for traumatic childhood experiences (Table 1). Additionally, nonremitters were more likely to have a current diagnosis of one or more psychiatric conditions and were more likely to report having taken an SSRI during the year leading up to the STEP trial, although the latter difference was not significant.

**Figure 1:**
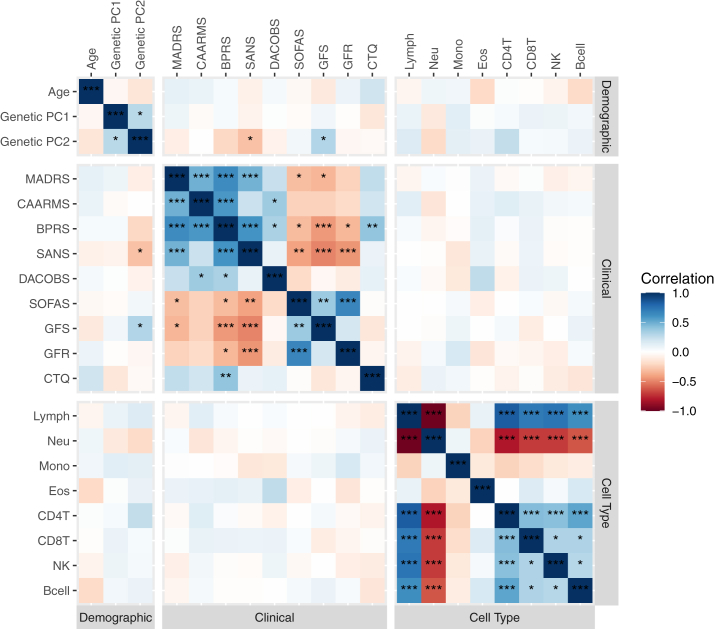
Pearson’s correlations between baseline demographic variables, clinical scores, and methylation-derived cell proportions. *p*_BH_ Values are represented as follows: ∗∗∗*p*_BH_ < 5 × 10^−4^, ∗∗*p*_BH_ < .005, ∗*p*_BH_ < .05. BPRS, Brief Psychiatric Rating Scale; CAARMS, the Comprehensive Assessment of At-Risk Mental States; CD4T, CD4+ T cell; CD8T, CD8+ T cell; CTQ, childhood trauma questionnaire; DACOBS, Davos Assessment of Cognitive Biases Scale; Eos, eosinophil; GFR, Global Functioning Role; GFS, Global Functioning Social; Lymph, lymphocyte; MADRS, Montgomery–Åsberg Depression Rating Scale; Mono, monocyte; Neu, neutrophils; NK, natural killer cell; *p*_BH_, Benjamini-Hochberg–adjusted *p*; PC, principal component; SANS, Scale for the Assessment of Negative Symptoms; SOFAS, Social and Occupational Functioning Assessment Scale.

Analysis of the correlation between the clinical assessment scores showed a high degree of correlation between most assessments (Figure 1). In particular, total MADRS and BPRS scores showed significant correlations with all other clinical measures assessed, including each other, while the CTQ score was only significantly correlated with the BPRS score and no other clinical measure.

### Baseline Lymphocyte Proportions Predict Remission of UHR Status

**Figure 2:**
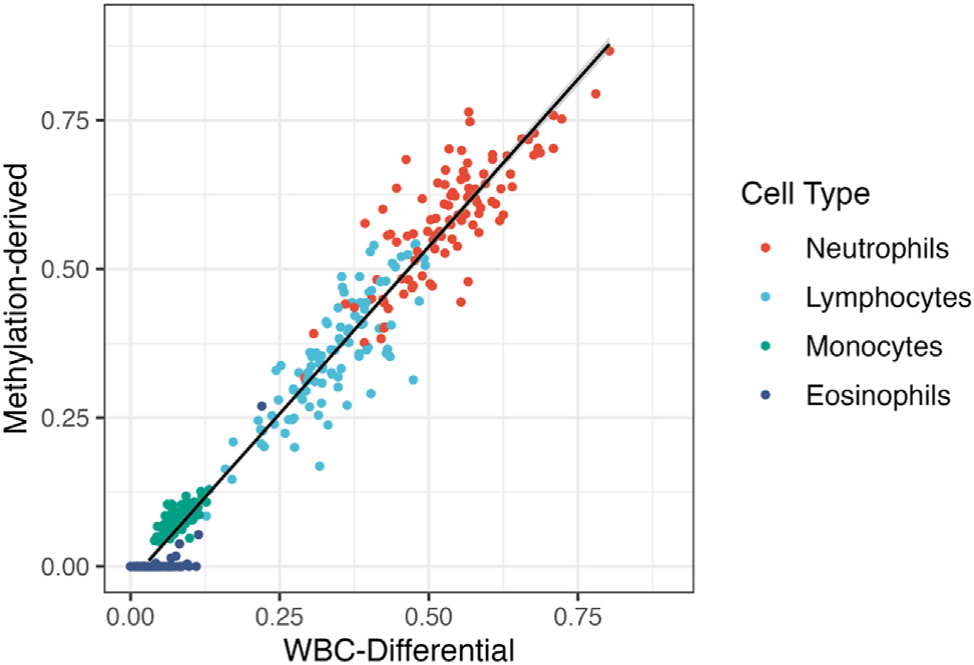
Methylation-derived cell-type proportions vs. WBCD-derived cell proportions from STEP 6- and 12-month samples. Cell proportions are significantly correlated, both across and within each cell type (all cell types, *R* = 0.98, *p* = 1.3 × 10^−300^; neutrophils, *R* = 0.82, *p* = 3.2 × 10^−26^; lymphocytes, *R* = 0.83, *p* = 1.1 × 10^−27^; monocytes, *R* = 0.69, *p* = 8.1 × 10^−16^; eosinophils, *R* = 0.64, *p* = 2.1 × 10^−13^). STEP, Staged Treatment in Early Psychosis; WBCD, white blood cell differential.

For the subset of participants with both a DNA methylation sample and a concurrent pathology test, methylation-derived cell-type proportions were validated against measured proportions from WBCDs. Methylation-derived lymphocyte subtypes were combined into a lymphocyte category, because individual lymphocyte subtypes were not available in the pathology results. The methylation-derived cell proportions were found to be highly correlated with the measured cell proportions (*R* = 0.98, *p* = 1.3 × 10^−300^) (Figure 2).

**Figure 3:**
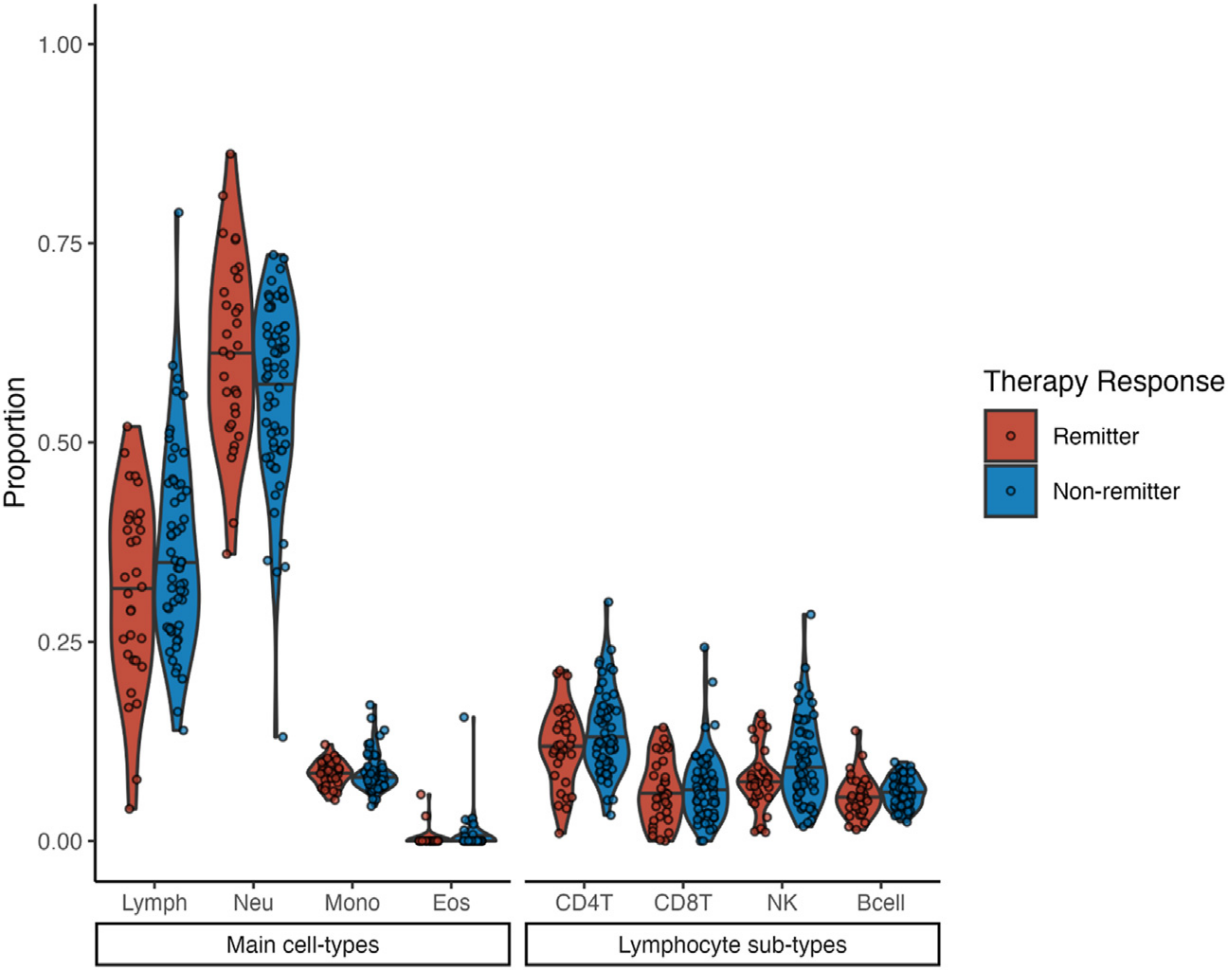
Methylation-derived cell-type proportions for remitters and nonremitters at trial baseline. CD4T, CD4+ T cell; CD8T, CD8+ T cell; Eos, eosinophil; Lymph, lymphocyte; Mono, monocyte; Neu, neutrophil; NK, natural killer cell.

An initial Wilcoxon-Mann-Whitney test did not identify any significant differences in methylation-derived cell proportions between remitters and nonremitters at baseline (Figure 3); however, cell-type proportions and DNA methylation are both affected by demographic variables such as age, sex, and ancestry (1,53,54). Therefore, methylation-derived cell proportions were tested for association with remission status using logistic regression analysis, which corrected for these demographic variables (Table 2). Remitters had significantly lower proportions of lymphocytes at baseline compared with nonremitters (odds ratio [OR], 0.46; 95% CI, 0.24–0.81; *p* = .011).

**Table 2:** Association of Methylation-Derived Cell Proportions at Trial Baseline With Remission Status

Cell Model	Cell Type	Demographic Covariates		Demographic and Clinical Covariates	
		Odds Ratio (95% CI)	*p*	Odds Ratio (95% CI)	*p*
Cell Categories	Lymphocytes	0.46 (0.24–0.81)	.011∗	0.23 (0.07–0.62)	.0092∗
	Monocytes	0.67 (0.38–1.13)	.15	0.60 (0.25–1.28)	.21
	Eosinophils	0.93 (0.44–1.48)	.77	1.27 (0.60–2.15)	.38
Lymphocyte Subtypes	CD4+ T cells	0.62 (0.28–1.27)	.20	0.20 (0.04–0.69)	.022
	CD8+ T cells	0.98 (0.55–1.79)	.93	0.97 (0.45–2.04)	.94
	Natural killer cells	0.54 (0.26–1.02)	.074	0.16 (0.03–0.57)	.012∗
	B cells	1.09 (0.58–2.05)	.79	1.55 (0.65–3.85)	.32
	Monocytes	0.66 (0.36–1.11)	.14	0.36 (0.10–0.94)	.066
	Eosinophils	0.86 (0.36–1.41)	.61	1.10 (0.36–1.97)	.80
NLR	NLR	1.99 (1.20–3.63)	.013	2.90 (1.37–7.46)	.012∗

		*R*^*2*^^a^	AIC	*R*^2^^a^	AIC

Cell Categories		0.16	117	0.52	82
Lymphocyte Subtypes		0.18	121	0.61	78
NLR		0.15	114	0.51	79

∗Significant at *p* < .0125.

AIC, Akaike information criterion; NLR, neutrophil-lymphocyte ratio.

a Tjur’s *R*^2^.

After adjusting for both demographic and clinical covariates (childhood trauma score, baseline MADRS score, and the presence of a psychiatric diagnosis at baseline), the association between lymphocytes and remission status increased (OR, 0.23; 95% CI, 0.07–0.62; *p* = 9.2 × 10^−3^). Sensitivity analyses confirmed that the effect was not driven by outlying samples, participant ancestry, or lifestyle factors (Figures S3–S7).

To determine whether the decrease in lymphocyte proportions was being driven by specific lymphocyte subtypes or was uniform across all lymphocytes, we tested the association of 4 lymphocyte cell subtypes with remission status (Table 2 and Table S7). None of the lymphocyte cell types were significantly associated with remission status when only demographic factors were adjusted for. However, natural killer cells were significantly lower in remitters after adjusting for clinical covariates (OR, 0.16; 95% CI, 0.03–0.57; *p* = .012).

The NLR was significantly higher in remitters relative to nonremitters when both demographic and clinical covariates were included (OR, 2.9; 95% CI, 1.37–7.46; *p* = .012) (Table 2 and Table S9) but was slightly over the multiple testing threshold when only demographic covariates were included (OR, 1.99; 95% CI, 1.20–3.63; *p* = .013). As with the lymphocytes and sublymphocytes, sensitivity analyses did not impact the conclusions.

MWAS analysis did not identify any significantly differentially methylated probes between remitters and nonremitters at baseline when cell-type proportions were corrected for. Likewise, no probes had a significant remission status–specific change in methylation between baseline and the 6-month time point (i.e., after the completion of step 2 in the trial). Similarly, no differentially expressed genes (DEGs) were identified for remission status at baseline or for the interaction between remission status and time point. Full details for both the MWAS and differential expression analyses can be found in Supplemental Results.

### Timing of Remission

Remission status was assessed at 2 steps during the trial, after step 1 (6 weeks) and after step 2 (6 months). At baseline, individuals who remitted at step 1 had nominally lower MADRS and CAARMS scores than step 2 remitters; however, these differences were not statistically significant after correction for multiple testing (Table 3).

**Table 3:** Baseline Demographic Variables and Clinical Characteristics of Stage 1 Remitters Compared With Stage 2 Remitters

Variable	Stage 1, *n* = 17	Stage 2, *n* = 14	*p* Value^a^	Missing, *n*
Age, Years	17.8 (4.0)	16.9 (3.1)	.40	0
Female	35.3%	50.0%	.48	0
European Ancestry	82.4%	85.7%	>.99	0
Preexisting Psychiatric Diagnosis	58.8%	42.9%	.48	0
Recent SSRI Use^b^	5.9%	7.1%	>.99	0
MADRS Total Score	11.9 (10.0)	18.3 (6.6)	.035	0
CAARMS Total Score	11.6 (7.4)	19.1 (7.0)	.0072	0
BPRS Total Score	36.0 (6.6)	40.4 (6.0)	.090	0
SANS Total Score	12.6 (7.4)	12.5 (11.5)	.50	0
SOFAS Score	59.4 (9.8)	61.4 (11.2)	.52	0
DACOBS Total Score	162.6 (30.5)	157.4 (19.2)	.53	0
Global Functioning: Social	7.0 (1.1)	7.0 (1.0)	.79	0
Global Functioning: Role	6.8 (1.2)	6.8 (1.9)	.55	0
Childhood Trauma Questionnaire Total Score	52.8 (8.5)	56.8 (13.9)	.77	0

Values are presented as mean (SD) or %.

BPRS, Brief Psychiatric Rating Scale; CAARMS, the Comprehensive Assessment of At-Risk Mental States; DACOBS, Davos Assessment of Cognitive Biases Scale; MADRS, Montgomery–Åsberg Depression Rating Scale; SANS, Scale for the Assessment of Negative Symptoms; SOFAS, Social and Occupational Functioning Assessment Scale; SSRI, selective serotonin reuptake inhibitor.

a Bonferroni-adjusted *p*-value threshold of 3.57 × 10^−3^.

b Reported use within the past 12 months.

**Figure 4:**
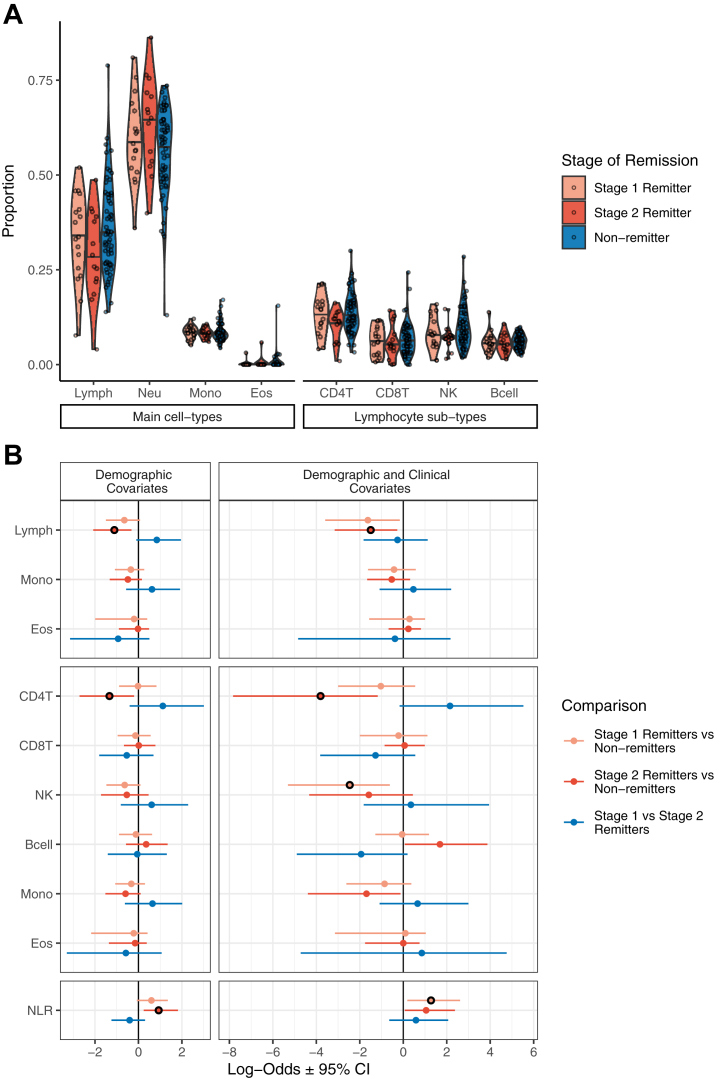
**(A)** Methylation-derived cell proportions at baseline for stage 1 remitters, stage 2 remitters, and nonremitters. **(B)** Pairwise logistic regression coefficients for stage of remission for each cell type tested. No cell types passed multiple-testing correction; associations that were nominally significant (*p* < .05) have been marked with black circles. CD4T, CD4+ T cell; CD8T, CD8+ T cell; Eos, eosinophil; Lymph, lymphocyte; Mono, monocyte; Neu, neutrophil; NK, natural killer cell.

Pairwise logistic regression was used to evaluate the association of cell-type proportions with the step of remission. No associations survived correction for multiple testing; however, as with the remitter versus nonremitter models, lymphocytes trended toward lower proportions in remitters of both steps relative to nonremitters, which decreased further when clinical covariates were accounted for (Figure 4 and Tables S12–S14). Despite total lymphocyte proportions being consistently lower in remitters at both steps, sublymphocyte cell types showed much less consistency between the steps, with CD4+ T cells in particular showing potential for step-specific differences in proportion. This may indicate that although total baseline lymphocyte proportion may be relevant for remission status, proportions of the sublymphocyte cell types may have potential relevance for time to remission within the remitter group.

An MWAS on the step of remission did not identify any significant probes at baseline; however, 11 probes showed a significant interaction between remission status and time point that was specific to remission at step 1 (relative to nonremitters), indicating potential heterogeneity between step 1 and step 2 remitters (Figure S8). Genes associated with these methylation probes appear to show stage of development–specific activity, with a significant enrichment in genes differentially expressed in the prenatal brain tissue at 21 weeks (Bonferroni-adjusted *p* = 8.54 × 10^−3^) (55). Differential expression analyses did not identify any DEGs for either step 1 or step 2 in the baseline-only analysis or for an interaction between the step of response and time point. Further details about both the MWAS and differential expression analyses can be found in Supplemental Results.

## Discussion

In this study, we identified a significant association between baseline proportions of methylation-derived WBCs and later remission of UHR status after treatment with psychosocial therapy. In particular, we found that lymphocyte proportions were significantly lower in future remitters relative to nonremitters and, furthermore, that this was not merely a reflection of differences in clinical profiles between the groups as it persisted after correcting for clinical measures.

Previous studies of WBCs in psychosis and SCZ have consistently identified differences in NLR in cases compared with healthy controls and, in particular, have identified higher NLR and therefore lower total lymphocyte proportions in SCZ and FEP cases (13, 14, 15, 16, 17). Here, we report lower NLR and higher total lymphocyte proportions for nonremitters compared with the less clinically severe remitters. This discrepancy is unlikely to be due to our cell data being methylation derived, because 2 previous studies used methylation-derived cell proportions to examine differences between SCZ and FEP cases relative to healthy controls (23,56). Neither of these 2 studies examined the total lymphocyte proportion; however, both reported lymphocyte subtypes with lower proportions in cases relative to controls, which is consistent with the previous NLR studies. Only 1 lymphocyte subtype, natural killer cells, was significant after multiple testing correction and only when both demographic and clinical covariates were included. However, the direction of effect for 3 of our 4 lymphocyte subtypes was the same as for our total lymphocyte proportion, suggesting that the association between lymphocytes and remission status is not primarily due to any one subtype.

The difference in direction of effect between our results and previous findings can be attributed to several reasons. First, previous studies compared diagnosed SCZ or FEP cases with healthy controls (13, 14, 15, 16, 17), whereas our study is a within-cohort comparison of individuals who all initially met the criteria for being UHR for psychosis, and therefore there is no comparison to healthy control individuals. While methylation data may be available for potential control groups from data repositories, methylation is highly susceptible to batch effects, and therefore any comparisons for which the grouping of interest is completely confounded with batch or collection group, as would be the case here, would not be appropriate (57).

Second, the criteria for UHR for psychosis are broad, and although they allow for successful enrichment of future psychosis cases relative to the general population, the majority of individuals identified do not go on to develop psychosis (58,59). Therefore, it may be that the differences between cell-type proportions are being driven by factors other than just liability for SCZ/FEP, especially given the prevalence of nonpsychotic mental disorders in the STEP cohort.

In addition to analyzing cell-type proportions, we also investigated differences in DNA methylation and gene expression with remission status. No methylation probes were associated with remission status; however, 11 probes showed an interaction between the step of remission and time point. Due to the small number of participants from each step who provided both baseline and 6-month samples, we suggest that this be taken largely as an indication of potential heterogeneity between step 1 and step 2 remitters. No DEGs were identified for remission status or the step of remission when cell proportions were included as covariates. As a cautionary note, sensitivity analyses in which cell proportions were not included in the remission status models led to a large number of genes being falsely identified as differentially expressed (Table S17). This highlights the importance of including cell-type information in gene expression analyses, where it is frequently overlooked due to the difficulty of obtaining accurate cell estimates.

A key limitation of this study is that the cell-type data is in the form of proportions and not counts, and this limits the interpretation of the results. Consistent with the idea that a higher NLR indicates increased systemic inflammation (7), the lower proportion of lymphocytes (as adaptive immunity cell types) relative to granulocytes and monocytes (innate immunity) in future remitters would suggest increased systemic inflammation in this group relative to nonremitters. However, it is also possible that despite having lower neutrophil and higher lymphocyte proportions than remitters, the nonremitters may still have higher total WBC counts and thus have higher counts for both neutrophils and lymphocytes than remitters. This is particularly likely, given that elevated total WBC counts have been observed in SCZ and psychotic disorders (2,60).

Another limitation is that the cell-type proportion data were estimated from DNA methylation and not measured directly. While the methylation-derived cell-type proportions for most cell types were highly correlated with measured proportion data in a subset of samples for which both were available, we were not able to validate the lymphocyte subtype proportions. Additionally, methylation-derived proportions for the low-abundance cell type of eosinophils were frequently estimated to be 0; these estimates were shown to be inaccurate for samples with directly measured WBC counts. This likely means that any association between eosinophil proportion and remission status is not accurately captured in our regression models. However, the extremely low abundance of these cell types means that the accuracy of associations between remission status and other cell types is not affected.

The findings of this study were limited by the small sample size. The size of the STEP clinical trial was established based on expected effect sizes in the clinical assessments, and the current DNA methylation and gene expression analyses were a secondary research add-on. In a simplified example using the baseline samples only, we had power to detect a true difference between means of 1.3 times the SD at a relatively nonstringent genome-wide significance threshold of 5 × 10^−6^. While this does not account for the multi-time point, interaction-based methods used for the MWAS and DEG analyses, this does indicate how these analyses were only powered to detect very large effect sizes. This lack of power not only reduces the ability to identify potential associations between our omic datasets and remission status but also limits the investigation of effects specific to the step of remission, which may represent a clinically relevant source of heterogeneity within the remitter group.

### Conclusions

Given the small sample size, our results should be seen as preliminary indications of the potential importance of WBCs for further stratification of psychosis risk in UHR individuals, with any conclusions being dependent on replication of these results in independent cohorts. Replication studies would also benefit from assessing whether the association of WBC with remission is solely a predictor of response to treatment or if it may predict the course of attenuated psychotic symptoms regardless of whether treatment is received. Additionally, given the relative ease and cost-effectiveness of obtaining WBC counts from standard pathology testing, the current results provide an evidence base to support the collection of this cell-type information as standard practice in future UHR clinical trials. The results of our study are made available for inclusion in meta-analyses.

## Supplementary Material

Supplemental Methods, Results, Figures S1–S9, and Tables S1–S2

Tables S3–S17

Key Resources Table
