# Supplementary material for: White Blood Cell Proportions Are Associated With Response to Psychosocial Therapy in Young People at Ultra-High Risk for Psychosis
Source: Biol Psychiatry Glob Open Sci. 2025 Jun 3;5(5):100546. doi: 10.1016/j.bpsgos.2025.100546 (PMC12281354; doi:10.1016/j.bpsgos.2025.100546)
Supplement: Supplemental Methods, Results, Figures S1–S9, and Tables S1–S2 [file mmc1.pdf]

SUPPLEMENTARY INFORMATION

White Blood Cell Proportions Are Associated With Response to Psychosocial Therapy in Young People at Ultra-High Risk for Psychosis

Barker *et al.*

Table of Contents

Supplementary Methods *3*

Genome-wide genotyping **3**

DNA Methylation **4**

Preparation of DNA Methylation samples 4

QC of DNA Methylation data 5

Methylome-Wide Association Study 6

Gene Expression **6**

RNA sequencing 6

RNA Mapping and QC 7

Differential Expression Analyses 7

Logistic Regression **8**

Logistic regression of remission status on cell proportions 8

Logistic regression of timing of remission on cell proportions 9

Supplementary Results *10*

Sensitivity analyses for logistic regression on cell proportions **10**

Removal/Winsorisation of outliers 10

Ancestry-restricted logistic regression on cell proportions 12

BMI 14

Smoking Exposure 15

Self-reported substance use 16

Methylome-Wide Association Study **19**

Differential Expression Analysis **21**

Supplementary References *22*

Supplementary Methods

Genome-wide genotyping

Genotyping was performed using the Illumina Infinium Global Screening Array (GSAMD) on DNA derived from either saliva (n=193) or whole blood (n=15). Initial quality control was performed per batch using PLINK v1.9(1). Samples were removed if they had high missingness (>10% of SNPs). SNPs were removed if they had high missingness (>10% of samples), minor allele frequency (MAF) less than 0.01, or deviated from Hardy-Weinberg equilibrium (p-value $< 1x10^{-6}$). Samples were imputed to the Haplotype reference consortium (HRC) (Version r1.1 2016). Imputed SNPs were removed if they had an imputation INFO score less than 0.8, MAF less than 0.01 or deviated from Hardy-Weinberg equilibrium. Genetic sex was imputed using PLINK and compared to participant's reported gender. Participants were assigned to broad ancestry groups based on principal component analysis of genetic similarity to predefined sets of individuals from the 1000 genomes project (Supplementary Figure 1) (2). Genetically-inferred ancestry was compared to reported countries of birth for participants and their parents, where available. Genetic relatedness of participants was estimated for all samples, as well as within genetic-ancestry groups, using GCTA(3). Duplicated samples (identified using genetic relatedness) were removed, as was one member of a pair of confirmed full-siblings. The sibling with less missing data was retained.

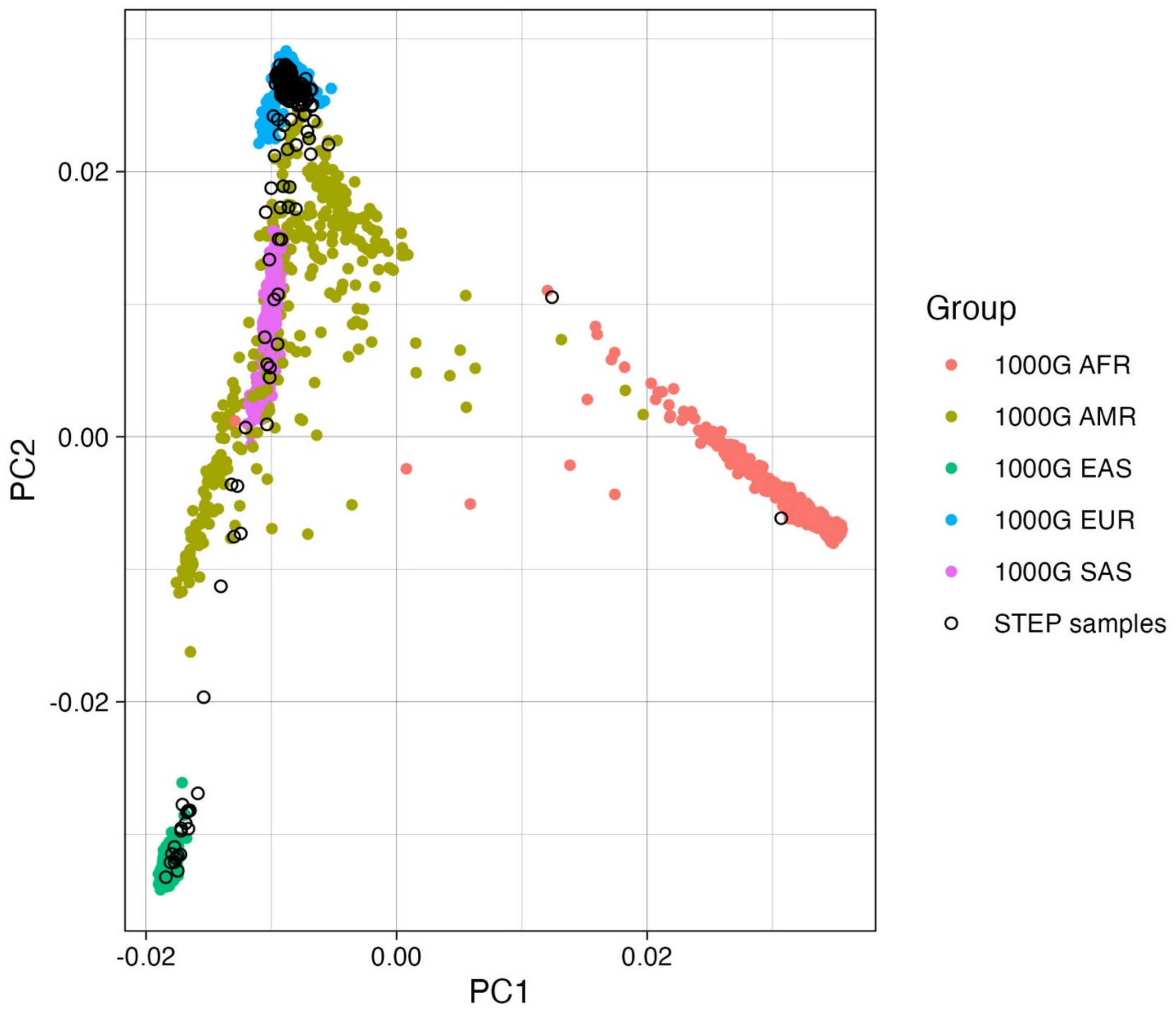

Supplementary Figure 1. Genetic PCs for STEP genotypes as projected onto the 1000 Genomes reference genotypes. Abbreviations: 1000G = 1000 Genomes, AFR = African, AMR = Admixed American, EAS = East Asian, EUR = European, SAS = South Asian.

DNA Methylation

Preparation of DNA Methylation samples

DNA concentration was quantified using the Take3™ Micro-Volume Plate on the Epoch™ Microplate Spectrophotometer (BioTek Instruments, Inc.) and samples with low concentration (< 25 ng/µl) were re-extracted. Bisulfite conversion was then performed with the Zymo EZ-96 DNA Methylation™ Kit Zymo Research, Irvine, CA, USA) and samples with enough converted DNA concentrations (>19 ng/µl) were taken forward to be assayed.

12 samples were excluded from the methylation assay. One sample was excluded due to failing bisulphite conversion and there was insufficient sample left for a re-run. All other exclusions were of duplicate samples, as only one of the duplicates was required.

Methylation levels were measured using the Illumina Infinium® MethylationEPIC BeadChip Assay. The samples were run in three batches of 96 samples, with the remaining samples run across two partial plates with samples from another study. Sample location on the plates was randomised, and human DNA controls and plate replicates were included.
Illumina Genome Studio v1.0 software was used to check the quality of the methylation assay output. All but two samples passed at a threshold of >860 000 site captured, and the majority of samples were very high quality.

QC of DNA Methylation data

QC and normalisation were performed with the R package Meffil, using recommended parameters(4). Samples were removed if they had dye bias, low methylation quality, sex mismatches, or had failed any of the pre-sequencing checks. Methylation probes were removed if they had poor detection scores. Probes displaying cross-reactivity or overlap with SNPs or repetitive elements were also removed, as recommended elsewhere(5). After QC, 351 samples and 779,503 probes remained.

Given the longitudinal study design, each participant may have contributed multiple samples. Methylation samples were, therefore, checked for identity against each other and against participant genotypes using the 59 SNPs on the EPIC array. Mislabelled samples were identified and either corrected or removed, depending on if the correct identity could be found and verified.

DNA methylation is measured as a per-site average of methylated to unmethylated probes within the sample, meaning that, in a whole blood sample, this will have been calculated using all the leukocytes present. Many DNA methylation sites show high cell-type specificity and, therefore, cell-type specific methylation profiles can be leveraged to estimate the proportions of individual cell-types within a mixture. Cell-type proportions were estimated using the Houseman method(6) as implemented in the *meffil* package. Cell-specific methylation profiles from Reinius et al.(7) were used as the reference dataset.

Methylome-Wide Association Study

DNA methylation samples were included if they met the criteria outlined in the main text for inclusion in the cell-proportion analysis.

Probes associated with remission status were identified using mixed-effect models as implemented in *R* packages *lme4* and *lmerTest*(8, 9). Sex, age, two genetic PCs, methylation-derived cell-type composition and methylation-predicted smoking exposure were included as covariates, along with technical covariates that the *meffil* normalisation report indicated were not fully normalised out (array row as fixed effect; array slide as random effect). Probes with a Benjamini-Hochberg-adjusted p-value less than 0.05 were considered significant.

MWAS were first performed in baseline samples to identify probes that may be associated with response. To capture probes with remission-status specific changes over time, a separate MWAS was then performed in a combined dataset of both baseline and 6-month samples, with timepoint included as an additional covariate and in an interaction term with remission status. Participant was fitted as a random effect, to account for the repeated-measures design.

As remitters may have experienced remission at either stage 1 or stage 2 of the trial, MWAS were also performed for stage of remission using the same methods described above. Non-remitters were used as the reference group to which stage 1 remitters and stage 2 remitters were compared.

Phenotypes previously associated with significant probes were identified using the MRC-IEU EWAS catalog (10). Probes were annotated to genes using the infinium MethylationEpic manifest. Tissue enrichment analyses were performed with the GENE2FUNC tool in FUMA(11) against GTEX V8 and BrainSpan tissue samples(12, 13).

Gene Expression

RNA sequencing

Whole blood was collected in PAXgene tubes and extracted using the PAXgene Blood RNA kit (Qiagen). 5ul aliquots of each sample were used to assess RNA concentration and integrity with Agilent's EPOCH and Bioanalyzer. Samples were prepared for sequencing using Stranded Total

RNA Ribo-zero plus prep kits (Illumina) followed by Paired End sequencing on Novaseq 6000 S4 v1.5 2x100bp flowcells (Illumina).

RNA Mapping and QC

Initial processing of sequenced reads was performed using fastp(14), which removes empty reads and trims any adapters detected on the reads. Reads were mapped to the GENCODE human genome primary assembly (v38) using STAR(15). The non-default ENCODE mapping parameters were used and two-pass mapping was performed, in order to better account for reads spanning unannotated splice junctions. Mapping quality was high for all samples (>80% uniquely mapping reads). Reads were then quantified using Rsubread's featureCounts(16), requiring both ends of a fragment to be mapped.

RNA samples were checked for identity against each other and against participant genotypes using SNVs called via GATK4 v4.2.1.0's 'RNAseq short variant discovery (SNPs + Indels)' best practices workflow(17).

Differential Expression Analyses

Baseline and 6-month gene expression data were included from participants who completed stages 1 and 2 of the trial, provided they also had DNA-methylation-estimated cell proportions for the same timepoint and genetically-inferred ancestry information available. One baseline sample was excluded due to taking an antidepressant in the 30 days prior to sample collection.

Differential expression analysis was performed to identify genes associated with remission status. Pre-filtering of genes was performed using the *filterByExpr* function in the *edgeR* package, which removes genes for which significance of differential expression cannot be accurately assessed due to consistently low expression in a high number of samples (as determined by the design matrix)(18). Differential expression analysis was then performed using a standard *limma-voom* workflow(19), with sex, age, sequencing pool and plate, two genetic PCs and methylation-derived cell-type compositions included as covariates. Genes with a Benjamini-Hochberg-adjusted p-value less than 0.05 were considered to be differentially expressed.

Differential expression was first performed in baseline samples to identify genes that may be predictive of remission. To capture genes with remission-status specific changes over time, DEG analysis was also performed in the combined dataset of baseline and 6-month samples. The

limma-voom workflow described above was used, with three adjustments: participant was fitted as a random effect, to account for the repeated-measures design; timepoint was included as an additional covariate; and an interaction term was included for timepoint and therapy response.

Remitters may have experienced remission at either stage 1 or stage 2 of the trial. To identify DEGs that may be specific to stage of remission, differential expression analysis was performed on baseline samples, 6-month samples, and on the combined set of baseline and 6-month samples using the same methods described above. Non-remitters were used as the reference group to which stage 1 remitters and stage 2 remitters were compared.

Logistic Regression

Logistic regression of remission status on cell proportions

Logistic regression was used to assess the association between remission status and methylation-derived cell proportions for WBCD-validated cell-types (Lymphocytes, Monocytes, Neutrophils and Eosinophils). As the methylation-derived cell data is proportionate in nature, only three of the four main cell-types are required to capture the total cell-type proportion distributions. Due to this, and the high level of correlation this induces between lymphocytes and neutrophils (the two most abundant cell types estimated; R = -0.97), only lymphocyte, monocyte and eosinophil proportions were included. Cell proportions were mean-centred and scaled by the standard deviation, to increase interpretability of results.

Two sets of covariates were fit, the first including only demographic covariates and the second including both demographic and clinical covariates, to account for any potential confounding effects of clinical variables on both cell proportions and remission status.
Demographic covariates were sex, age and the first two genetic principal components (PCs), which represent genetically-inferred ancestry. Clinical covariates included total childhood trauma score (CTQ), total MADRS score at baseline and whether a participant had an established psychiatric diagnosis at baseline (yes/no).

Demographic covariates:

```
Remission Status ~ Sex + Age + Genetic PC1 + Genetic PC2 + Lymphocytes + Monocytes
+ Eosinophils
```

Demographic + Clinical covariates:

```
Remission Status ~ Sex + Age + Genetic PC1 + Genetic PC2 + Baseline MADRS + CTQ +
Has Psychiatric Diagnosis (Yes/No) + Lymphocytes + Monocytes + Eosinophils
```

Methylation-derived cell-type proportions were available for four lymphocyte cell sub-types: CD4+ T-cells, CD8+ T-cells, B cells and natural killer cells. To determine which lymphocyte sub-types might be driving the association with remission status, logistic regression was performed using the covariate sets described above, with the lymphocyte sub-types in place of lymphocytes.
Finally, the log neutrophil-lymphocyte ratio was calculated as log(Neutrophils/Lymphocytes) for all samples and assessed for association with remission status as described above.

As the cell-type proportions are highly inter-correlated, we calculated a multiple-testing corrected p-value threshold of $p < 0.0125$. This corrects for 2 sets of covariates and 2 sets of 'independent' cell-types, reflecting that the data used in the logistic regression with the NLR have already been tested.

Logistic regression of timing of remission on cell proportions

Remitters may have experienced remission at either step 1 or step 2 of the trial. To assess whether step of remission was associated with cell-type proportions, pairwise logistic regressions comparing step 1 remitters, step 2 remitters and non-remitters were performed using the cell types and models described above.

A multiple-testing corrected p-value threshold of $p < 6.25 \times 10^{-3}$ was used to declare significance. This corrects for 2 sets of covariates, 2 sets of 'independent' cell-types and 2 independent pairwise regressions, as the third regression is a linear combination of the other two.

Supplementary Results

Sensitivity analyses for logistic regression on cell proportions

Removal/Winsorisation of outliers

To ensure the association between lymphocyte cell proportions and remission status was not being driven by the presence of one sample with exceedingly high lymphocytes and low neutrophils, we removed the outlier sample and repeated the logistic regression analyses. Further, to ensure the observed associations were not being driven by extreme outliers more generally, we performed a 97.5% winsorisation on cell proportions(20, 21), after mean-centring and scaling by SD (i.e. as prepared for the logistic regression;Supplementary Figure 2).

Removal of the outlier sample did not meaningfully affect the estimates or interpretation of the association between cell proportions and remission (Supplementary Figure 3), and nor did winsorisation of extreme cell proportions. Therefore, the observed associations between remission status and baseline cell proportions is unlikely to be driven by outlier samples.

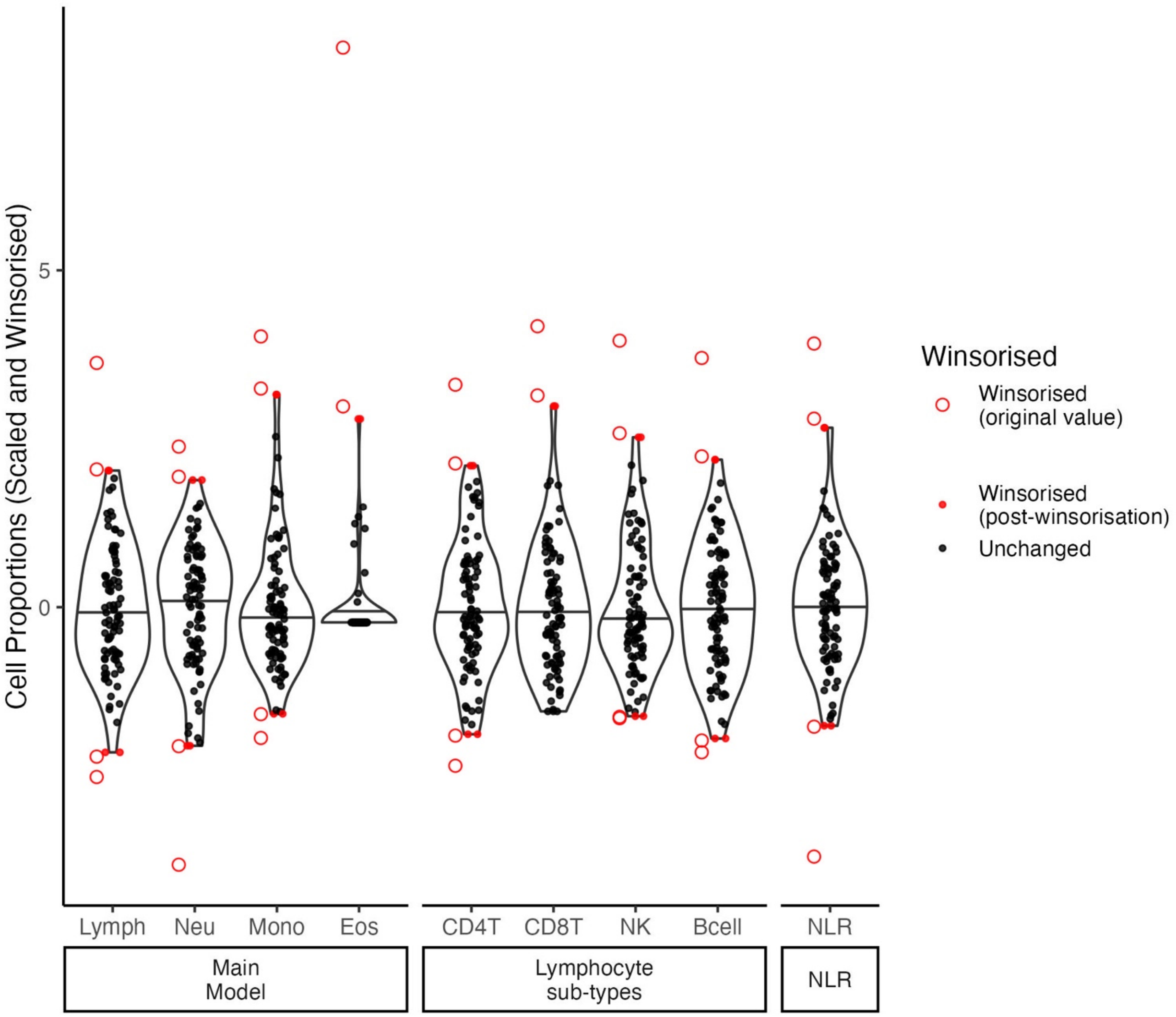

Supplementary Figure 2. Mean-centred and SD-scaled proportions for each cell-type. Samples affected by winsorisation are shown in red, with the pre-winsorised values represented by large unfilled circles and the post-winsorisation values by small red dots. Points unaffected by winsorisation are represented only once, as small black dots.

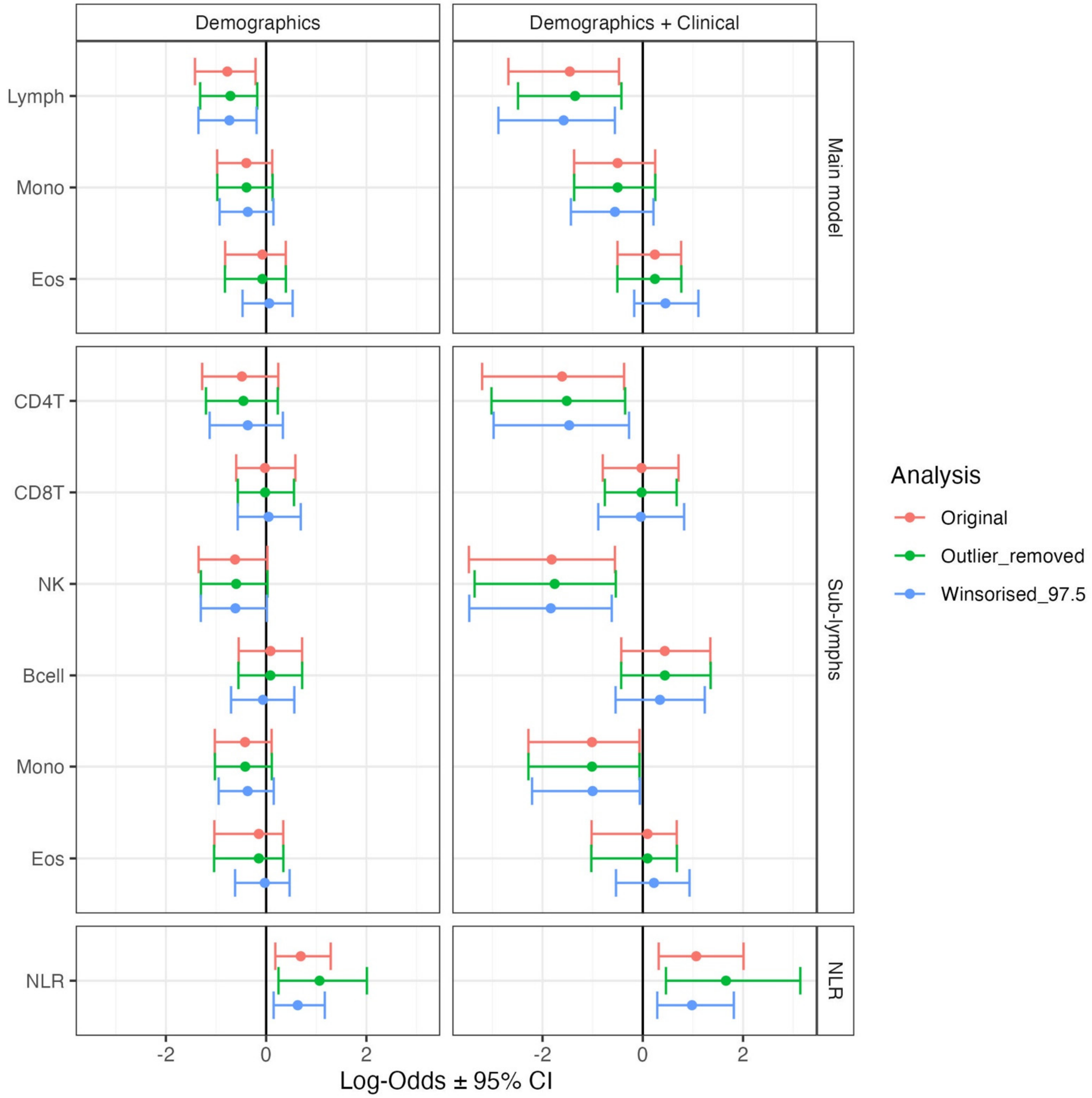

Supplementary Figure 3. Comparison of results from the original logistic regression in the main text, to the same analyses repeated after removing the outlier sample or after performing a 97.5% winsorisation of extreme values.

Ancestry-restricted logistic regression on cell proportions

This is a multi-ancestry cohort, consisting predominantly of individuals of genetically-inferred European ancestry. Although the proportion of non-European participants is not significantly different between remitters and non-remitters, we nonetheless observed nominally significant association between genetic PCs and remission status in our main logistic regressions

(supplementary tables 6-9). Additionally, the reference dataset used to estimate cell proportions in sample methylation data was comprised solely of participants of European ancestry. As such, although genetic PCs were included as covariates in the logistic regressions, participant ancestry may still bias our results. To account for this, we performed logistic regression on an ancestry-restricted subset of samples with genetically-inferred European ancestry.

There were 71 participants inferred to be of European ancestry, of which 26 were remitters and 45 non-remitters. Restricting the analyses to these participants did not meaningfully change the association between remission status and cell proportions and, further, resulted in slightly strengthened odds-ratios of association for both lymphocytes and the NLR (Supplementary Figure 4).

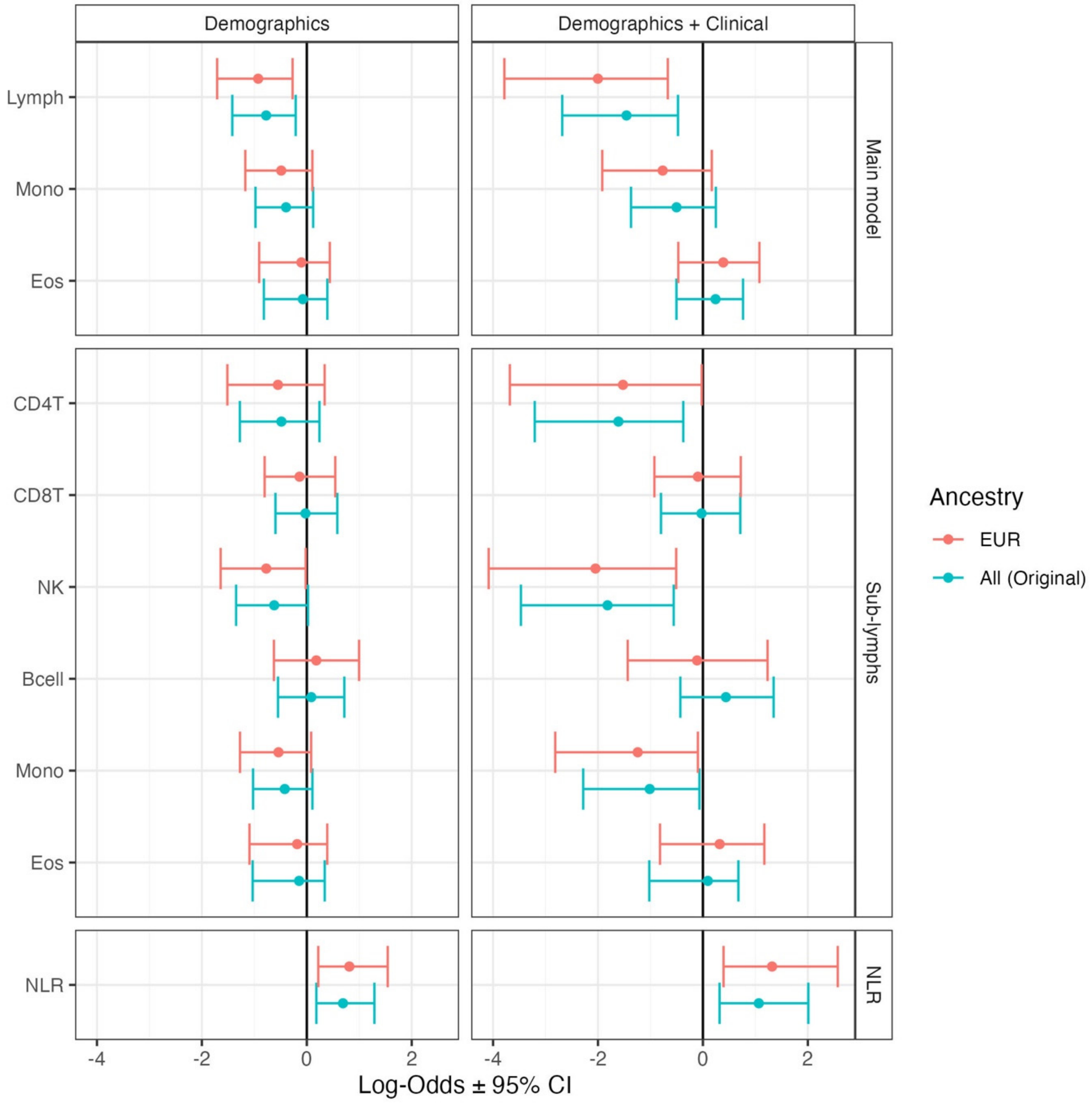

Supplementary Figure 4. Comparison of results from the original logistic regression in the main text, to the ancestry-restricted analyses performed using only participants with genetically-inferred European ancestry (n remitters = 26, n non-remitters = 45).

BMI

82 participants (30 Remitters) had non-missing height and weight measurements at Baseline, which were used to calculate BMI. Logistic regression was performed on the samples with non-missing BMI measures, using the same covariates as before with the addition of BMI. Due to the missing samples potentially biasing any comparison of the original results to the BMI-included results, the original models were also re-run using only the samples with non-missing BMI measures. BMI was not significantly associated with remission status for any of the models tested, and nor did it have any meaningful effects on the association between cell proportions and remission status (Supplementary Figure 5).

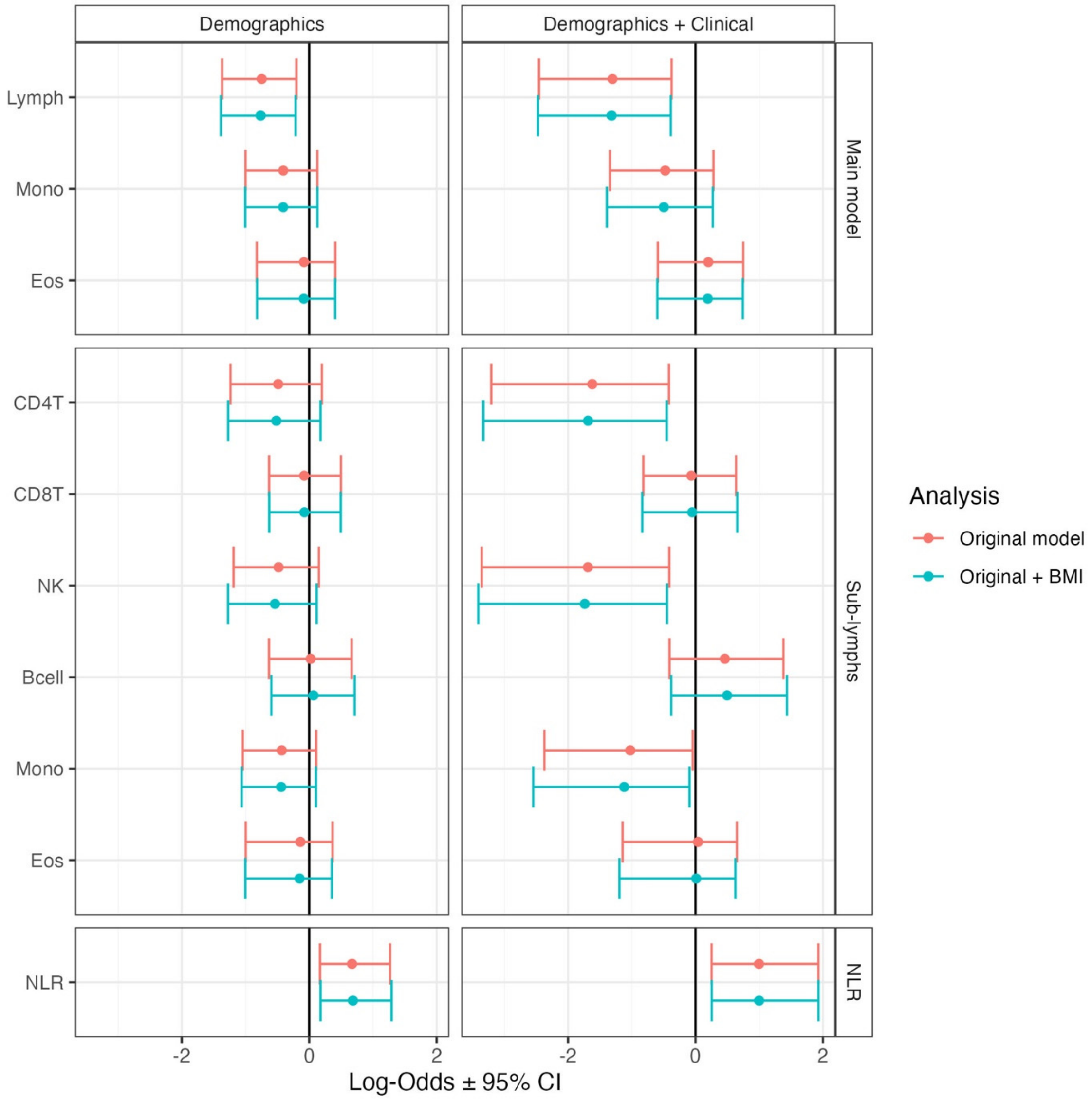

Supplementary Figure 5. Comparison of results between the original logistic regression models in the main text and ones in which BMI was included as an additional covariate. To account for samples with missing BMI measurements, both the 'Original' and 'Original + BMI' regressions shown here were performed using only participants with non-missing BMI measures (n remitters = 30, n non-remitters = 52).

Smoking Exposure

Tobacco smoking exposure was estimated using methylation profile scores (MPS). Smoking MPS were generated using OSCA, with predictors from McCartney, et al and were validated using self-reported tobacco use behaviour collected at Baseline using the World Health Organisation's Alcohol, Smoking and Substance Involvement Screening Test (ASSIST). Self-reported 'Never' smokers (n=43) had significantly lower smoking MPS than self-reported 'Daily or more' (n=27) smokers ($p = 1.5 \times 10^{-4}$), validating the use of the smoking MPS as a quantitative proxy for smoking exposure. To evaluate whether smoking was affecting the association between cell-type

proportions and remission status, logistic regressions were then performed using the original demographic and clinical covariates plus the smoking MPS. The smoking MPS was not significantly associated with remission status, and including the smoking MPS had minimal effect on the association between cell proportions and remission status (Supplementary Figure 6).

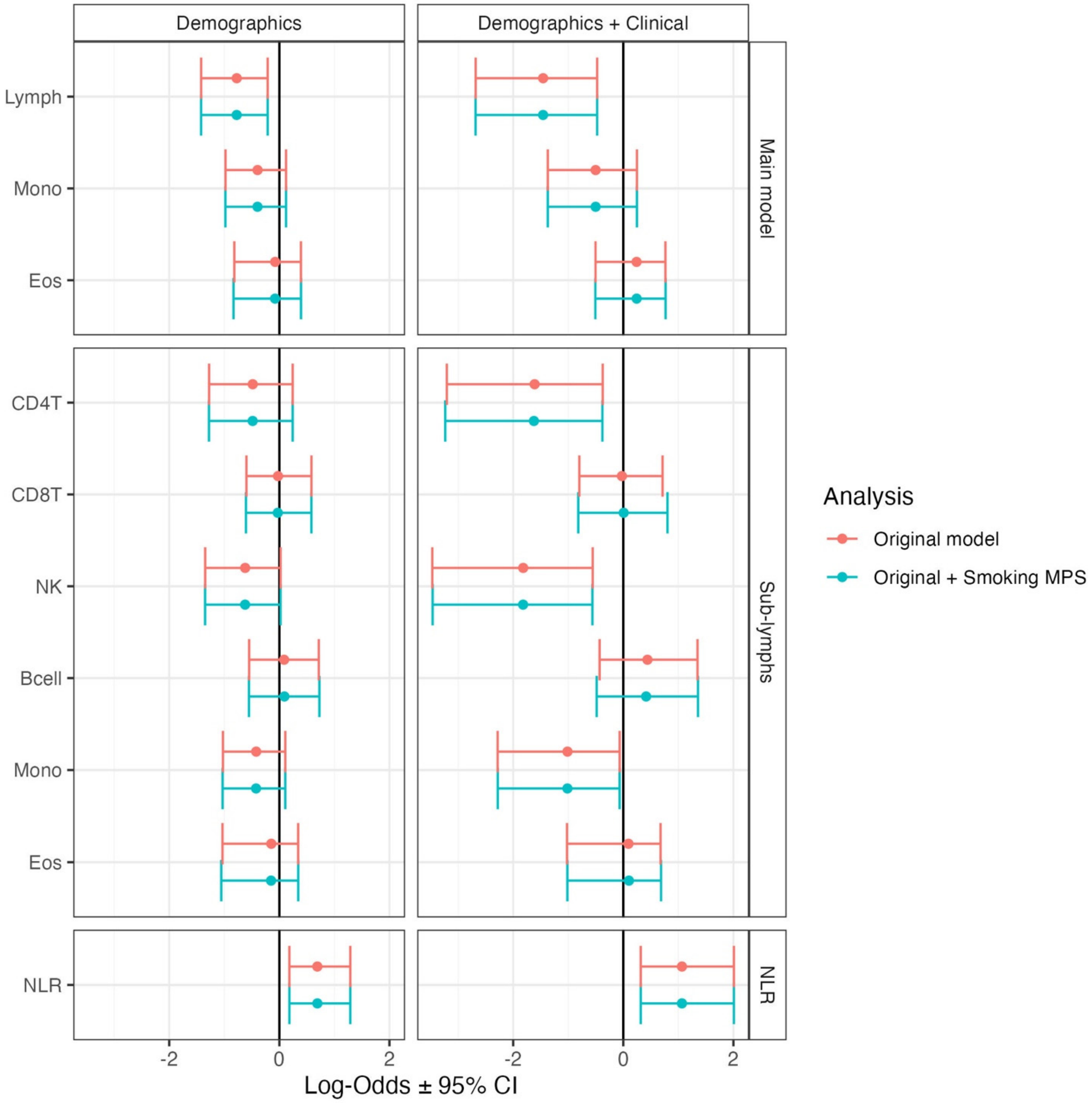

Supplementary Figure 6. Comparison of results from the original logistic regression in the main text, to the same analyses repeated with the inclusion of Smoking MPS as an additional covariate.

Self-reported substance use

STEP participants were asked to provide information on their non-prescription substance use behaviours using the World Health Organisation's Alcohol, Smoking and Substance Involvement

Screening Test (ASSIST). In total, 86 participants had non-missing baseline entries for substance use (n Remitters=28), with the exception of sedative use, which was missing an additional non-responder participant. Substance use frequency was converted into a pseudo-quantitative measure of days per month as follows: Daily = 30 days/month, Weekly = 4.3 days/month, Monthly = 1 days/month, 'Once or Twice' = 0.5 days/month, Never = 0 days/month. This was performed in order to better reflect the difference in usage frequency between the categories. Samples with missing substance use entries were excluded and Logistic regressions were performed per substance, using the original model with the substance use frequency included as an additional covariate. As a comparison, the original model was also re-run in this reduced subset of samples.

The inclusion of self-reported substance use frequency did not meaningfully affect the estimate or interpretation of the cell proportion associations with remission status (Supplementary Figure 7). It should be noted that, in this down-sampled analysis, even the results for the 'original' model do not pass the established significant thresholds in the main paper. This likely due to a loss of power due to the reduction in sample size.

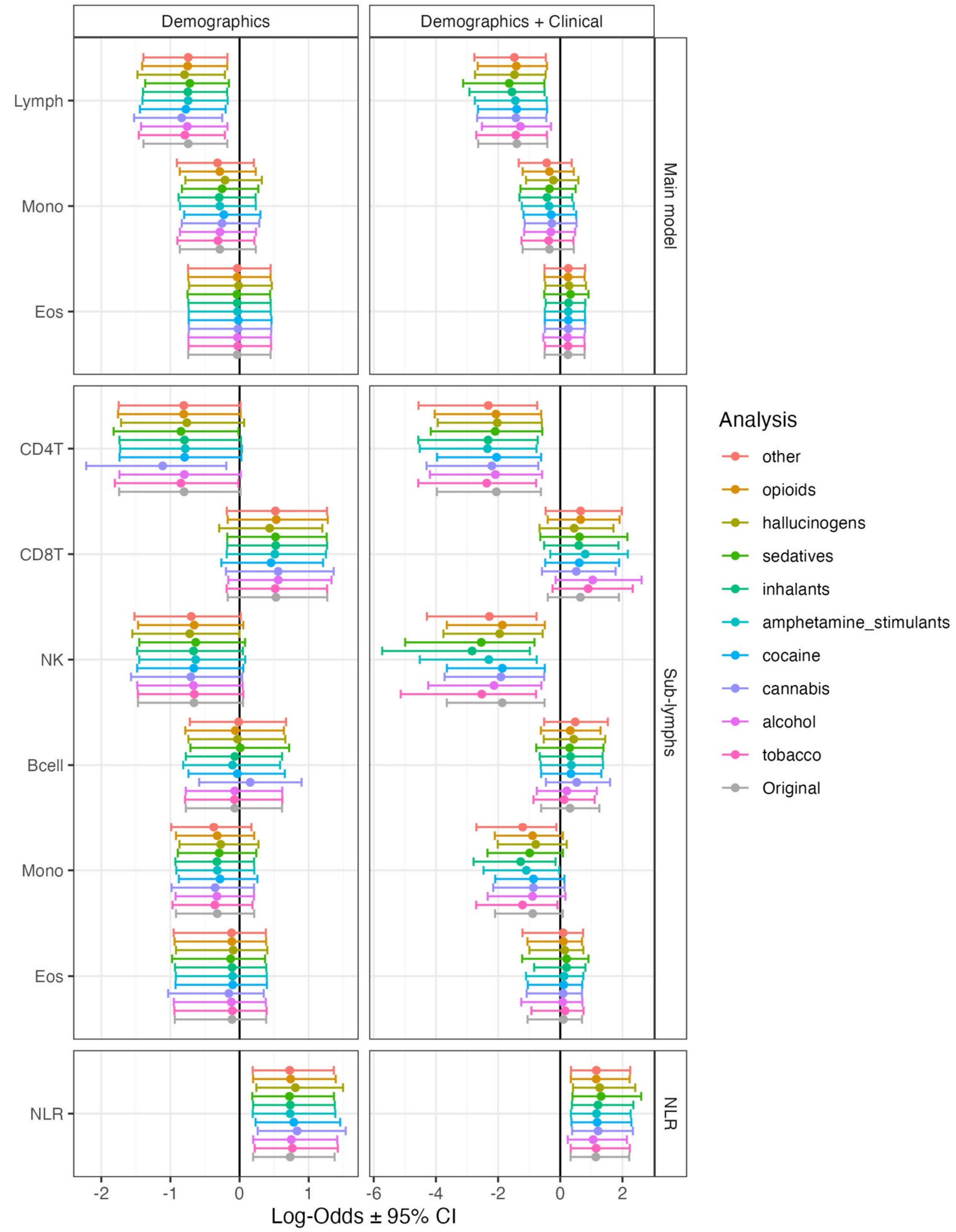

Supplementary Figure 7.Self-reported substance use. Comparison of association of cell proportions to remission status using the original covariates (in a subset of samples with non-missing ASSIST entries) to association results when substance use frequency is included as an additional covariate.

Methylome-Wide Association Study

Supplementary Table 1. Number of DNA-methylation samples at each timepoint for Remitters and Non-remitters. Participants who have provided both a baseline and a 6-month sample are already included in the per-timepoint count but have also been noted separately at the end of the end of the table

Timepoint	Remitter (Stage 1 Remitter)	Non-remitter	Total per timepoint
**Baseline**	31 (17)	60	91
**6-month**	16 (6)	65	81
**Participant has both timepoints**	15 (6)	53	68 per timepoint

91 Participants provided samples at baseline (31 Remitters, Supplementary Table 1), while 81 participants provided 6 month samples (16 Remitters). In order to identify probes that were associated with remission status, MWAS were performed on both baseline samples and on a combined dataset of baseline and 6-month samples. No probes were significantly associated with remission status at baseline and no probes showed a significant remission-status-specific change in methylation over time.

The psychosocial therapy portion of the trial consisted of two stages, with remission status assessed after each one. Given that participants who experienced remission during stage 1 required fewer treatments than those who remitted during stage 2 (6 weeks vs 24 weeks), and thus may have different underlying factors contributing to UHR status, grouping all remitters may reduce the ability to detect stage-specific effects of remission status. To account for this, MWAS were performed on stage of remission.
No probes were significantly associated with stage of remission at baseline, however 11 probes showed a significant interaction between remission status and timepoint that was specific to remission at Stage 1 (relative to Non-remitters; Supplementary Figure 8 & Supplementary Table 16).

A

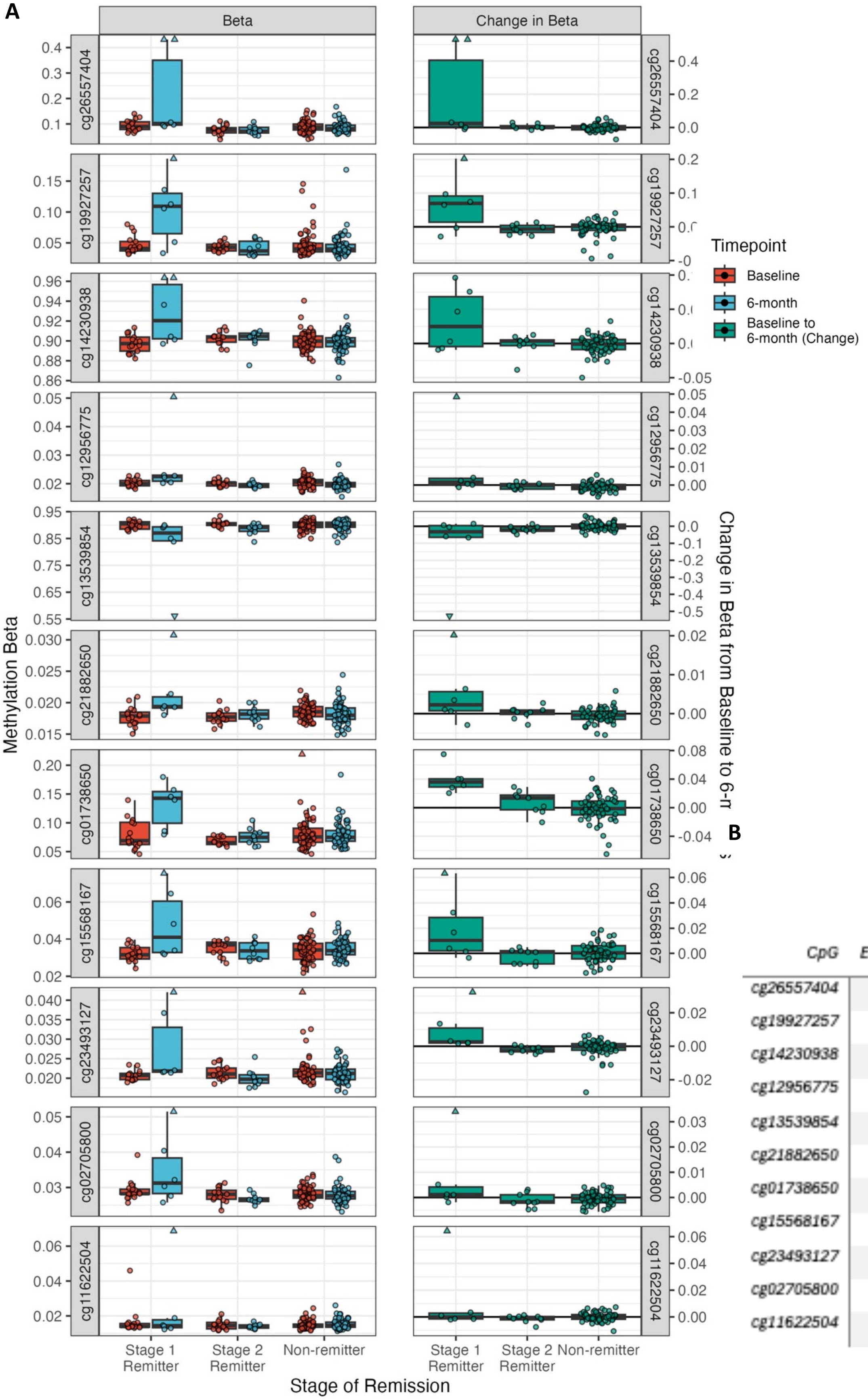

B

CpG	Estimate	Std_Error	FDR
cg26557404	0.2	0.029	1.88e-04
cg19927257	0.079	0.012	1.88e-04
cg14230938	0.035	0.006	4.00e-03
cg12956775	0.016	0.003	1.32e-02
cg13539854	-0.175	0.031	1.69e-02
cg21882650	0.006	0.001	1.69e-02
cg01738650	0.046	0.008	1.69e-02
cg15568167	0.02	0.004	4.54e-02
cg23493127	0.011	0.002	4.86e-02
cg02705800	0.01	0.002	4.86e-02
cg11622504	0.021	0.004	4.90e-02

Supplementary Figure 8. A)Normalised beta per timepoint, and change in normalised beta between timepoints, for CpGs that had a significant timepoint x Stage of remission effect. Methylation betas and beta-changes were winsorised for plotting purposes to mean(beta) ± 5xSD(beta). Points that were affected by winsorisation are represented by triangles. B) Results of MWAS for the Stage1 Remitters (vs Non-remitters) x Timepoint Interaction, also available in Supplementary Table 16.

These probes were annotated to 14 genes (Supplementary Table 16), all of which were recognisable by FUMA and used for tissue enrichment analyses(11). These genes were significantly enriched in DEGs expressed in prenatal brain tissue at 21 weeks (Bonferroni-adjusted p-value = $8.54x10^{-3}$) and in down-regulated DEGs of Heart Atrial Appendage (Bonferroni-adjusted p-value=0.01). Additionally, expression of these genes in brain tissues at various stage of pre- and post-natal development appears to show stage-of-development specific activity, with many of these genes being more highly expressed in prenatal brain tissue (Supplementary Figure 9)(12).

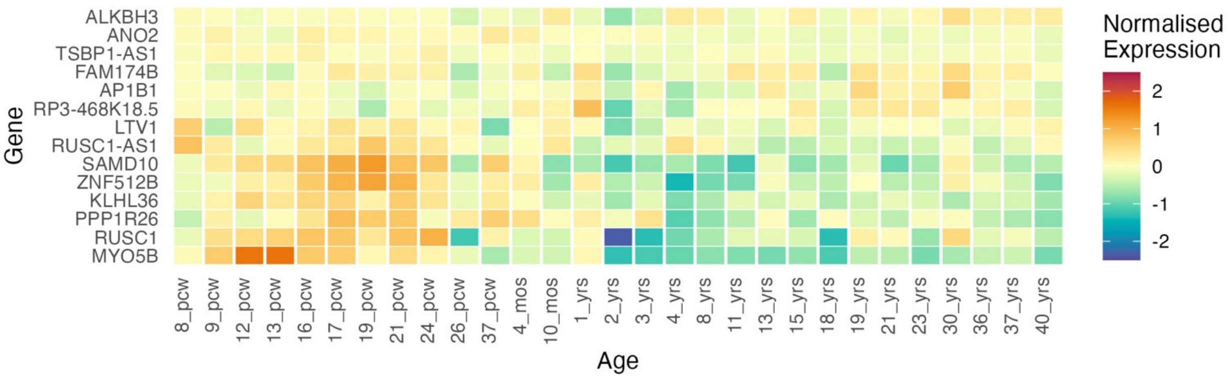

Supplementary Figure 9. Expression of genes with methylation probes associated with the interaction between stage of remission (stage 1) and timepoint in aggregated brain tissues across developmental stages.

Differential Expression Analysis

81 participants provided eligible trial baseline samples, of which 25 would go on to experience remission of UHR status after treatment with psychosocial therapy (Supplementary Table 2). 81 participants provided samples at 6 months, after the psychosocial therapy portion of the trial had ended. 57 of these participants provided both a baseline and 6-month sample, 11 of which were remitters.

Supplementary Table 2. Number of remitters and non-remitters used for differential expression analyses at each timepoint. Participants who have provided both a baseline and a 6-month sample are already included in the per-timepoint count but have also been noted separately at the end of the table.

Timepoint	Remitter (Stage 1 Remitter)	Non-remitter	Total per timepoint
**Baseline**	25 (11)	56	81
**6-month**	16 (7)	65	81
**Participant has both timepoints**	11 (3)	46	57 per timepoint

In order to identify genes that may be associated with, or involved in, remission of UHR status after treatment with psychosocial therapy, differential expression analyses were performed separately on samples taken at trial baseline and on a combined dataset of baseline and 6-month samples. No differentially expressed genes (DEGs) were found for remission status at baseline or in the combined analysis of baseline and 6-month samples. Likewise, no genes showed differential expression consistent with an interaction between timepoint and remission status.

As the remitter group was comprised of both Stage 1 and Stage 2 Remitters, differential expression analyses were also performed on stage of remission. No DEGs were identified for either stage 1 or stage 2 in the baseline-only analysis, and nor were any identified for an interaction between stage of response and timepoint.

Supplementary References

1. Purcell S, Chang C PLINK. 1.9 ed.
2. Auton A, Abecasis GR, Altshuler DM, Durbin RM, Abecasis GR, Bentley DR, et al. (2015): A global reference for human genetic variation. *Nature*. 526:68-74.
3. Yang J, Lee SH, Goddard ME, Visscher PM (2011): GCTA: a tool for genome-wide complex trait analysis. *Am J Hum Genet*. 88:76-82.
4. Min JL, Hemani G, Davey Smith G, Relton C, Suderman M (2018): Meffil: efficient normalization and analysis of very large DNA methylation datasets. *Bioinformatics*. 34:3983-3989.
5. Zhou W, Laird PW, Shen H (2017): Comprehensive characterization, annotation and innovative use of Infinium DNA methylation BeadChip probes. *Nucleic Acids Research*. 45:e22-e22.

6. Houseman EA, Accomando WP, Koestler DC, Christensen BC, Marsit CJ, Nelson HH, et al. (2012): DNA methylation arrays as surrogate measures of cell mixture distribution. *BMC Bioinformatics*. 13:86.
7. Reinius LE, Acevedo N, Joerink M, Pershagen G, Dahlen SE, Greco D, et al. (2012): Differential DNA methylation in purified human blood cells: implications for cell lineage and studies on disease susceptibility. *PLoS One*. 7:e41361.
8. Kuznetsova A, Brockhoff PB, Christensen RHB (2017): lmerTest Package: Tests in Linear Mixed Effects Models. *J Stat Softw*. 82:1-26.
9. Bates D, Mächler M, Bolker BM, Walker SC (2015): Fitting Linear Mixed-Effects Models Using lme4. *J Stat Softw*. 67:1-48.
10. Battram T, Yousefi P, Crawford G, Prince C, Sheikhali Babaei M, Sharp G, et al. (2022): The EWAS Catalog: a database of epigenome-wide association studies. *Wellcome Open Res*. 7:41.
11. Watanabe K, Taskesen E, van Bochoven A, Posthuma D (2017): Functional mapping and annotation of genetic associations with FUMA. *Nat Commun*. 8:1826.
12. Kang HJ, Kawasawa YI, Cheng F, Zhu Y, Xu X, Li M, et al. (2011): Spatio-temporal transcriptome of the human brain. *Nature*. 478:483-489.
13. Consortium G (2020): The GTEx Consortium atlas of genetic regulatory effects across human tissues. *Science*. 369:1318-1330.
14. Chen S, Zhou Y, Chen Y, Gu J (2018): fastp: an ultra-fast all-in-one FASTQ preprocessor. *Bioinformatics*. 34:i884-i890.
15. Dobin A, Davis CA, Schlesinger F, Drenkow J, Zaleski C, Jha S, et al. (2013): STAR: ultrafast universal RNA-seq aligner. *Bioinformatics*. 29:15-21.
16. Liao Y, Smyth GK, Shi W (2019): The R package Rsubread is easier, faster, cheaper and better for alignment and quantification of RNA sequencing reads. *Nucleic Acids Res*. 47:e47.
17. Auwera Gvd, O'Connor BD (2020): *Genomics in the cloud : using Docker, GATK, and WDL in Terra*. First edition. ed. Sebastopol, CA: O'Reilly Media.
18. Chen Y, Lun AT, Smyth GK (2016): From reads to genes to pathways: differential expression analysis of RNA-Seq experiments using Rsubread and the edgeR quasi-likelihood pipeline. *F1000Res*. 5:1438.
19. Law CW, Chen Y, Shi W, Smyth GK (2014): voom: Precision weights unlock linear model analysis tools for RNA-seq read counts. *Genome Biol*. 15:R29.
20. Dixon WJ (1960): Simplified Estimation from Censored Normal Samples. *Ann Math Stat*. 31:385-391.
21. Signorell A (2025): DescTools: Tools for Descriptive Statistics. R package version 0.99.60 ed.
